# Tests of rubber granules used as artificial turf for football fields in terms of toxicity to human health and the environment

**DOI:** 10.1038/s41598-022-10691-1

**Published:** 2022-04-23

**Authors:** Beata Grynkiewicz-Bylina, Bożena Rakwic, Barbara Słomka-Słupik

**Affiliations:** 1 Laboratory of Material Engineering and Environment, KOMAG Institute of Mining Technology, 44-101 Gliwice, Poland; 2 Faculty of Civil Engineering, Silesian University of Technology, 44-100 Gliwice, Poland

**Keywords:** Ecology, Environmental sciences, Risk factors, Chemistry, Engineering, Materials science

## Abstract

Rubber waste, in the form of granules of styrene butadiene rubber and ethylene-propylene-diene-monomer with a particle size of 0.5 to 4 mm, is broadly used for the construction of synthetic surfaces of sport fields. This method of recycling may be significantly limited due to the restrictions on polycyclic aromatic hydrocarbons (PAHs) content in rubber granules in the European Union since 2022. This also applies to the recommendations of the European Chemicals Agency in relation to the identification of other hazardous chemicals in this waste, including metal elements. The scope of the research included the identification of organotin compounds, PAHs content and 18 elements leached from recycled rubber granules in terms of substances harmful to human health and to natural environment. The research covered 84 samples of rubber granules collected from the surface of football pitches or supplied by recyclers in Poland. The test results showed an over-standard content of PAHs in rubber granules. This result confirms the need to develop alternative directions of rubber granules application: construction and hydro construction, reinforcing soil and roadsides, asphalt pavements, making retaining walls, anti-shock and anti-vibration slabs, soundproofing and damping screens, paving stones and landscaping elements.

## Introduction

Polymer waste, due to the increasing amount and long decomposition time, belongs to the group of wastes that are particularly problematic for the natural environment. Among them are recycling end-of-life car tires (ELTs) in a significant quantity. More than 3.1 million tons of tires are used annually in the EU, according to the European Tyre & Rubber Manufacturers Association^1^. According to: Council Directive 1999/31/EC of 26 April 1999 on the landfill of waste^2^, Directive 2000/53/EC of the European Parliament and of the Council of 18 September 2000 on end-of life vehicles^3^, “European Green Deal”^4^ and “The New EU Action Plan for the Circular Economy for a Cleaner and More Competitive Europe”^5^, activities should be taken to increase the use of secondary raw materials obtained from car tires. Material recycling carried out with the use of physical processes including cleaning, multi-stage mechanical grinding, screening of rubber material, separation of metal and textile parts is used^6–10^. After these processes, fragmented rubber materials: steel wires and textile cord are obtained. Depending on the degree of grinding, the rubber material can be used in various ways^11–16^. According to data from 2018, more than 1.5 million tons of car tires were processed in this way. This accounts for 83% of recycled tires^1^. Popular is the construction of sport fields with artificial turf made of rubber granules made from tires with a particle size of 0.5 to 4 mm^17^. Granules are a filling material ensuring the resilience of the surface and absorbs falls^18^. If the appropriate binder is selected, granules are used to make solid boards for the surface of sport fields and playgrounds. Apart from ELTs, other types of polymers from household appliances and the automotive industry are used in the construction of sport fields, but their amount did not exceed 2% of the granules used for the surface of sport fields, in exemplary 2015 year^17^. They were ethylene-propylene-diene monomer (EPDM) and thermoplastic elastomers: styrenic block copolymers (TPS), thermoplastic polyolefins (TPO), thermoplastic vulcanizates (TPV), thermoplastic polyurethanes (TPU), thermoplastic copolyester (TPC) and thermoplastic polyamides (TPA)^17,19^. The chemical composition of tires does not differ from the chemical composition of rubber granules obtained in the recycling process. The car tires granules contain 43% of SBR (styrene-butadiene rubber), 28% of carbon black, 2% of zinc oxide, 1% of sulphur and 6 to 8% of additives containing organic low-molecular substances (OLMS)^17^. OLMS are extender oils, vulcanization accelerators (mainly amine, thiuram and carbamate derivatives), antioxidants (most often phenolic derivatives), organic peroxides and others^17^. Literature data shows that in addition to the above-mentioned ingredients there may be chemicals that are toxic to human health and to environment in recycled tire granules used in the sport fields and playgrounds^18,20–32^. Their source may be raw materials of inadequate quality or impurities from the rubber vulcanization process^18,33^. These substances include polycyclic aromatic hydrocarbons (PAHs), heavy metals: lead (Pb), cadmium (Cd), arsenic (As) and mercury (Hg); phthalates and volatile organic compounds (VOCs). PAHs are classified according to the Regulation of the European Parliament and of the European Council No. 1272/2008^34^ as carcinogenic substances of category 1B. Benzo[*a*]pyrene belongs to mutagenic substances of category 1B and toxic to reproduction in category 1B, as well. Chrysene belongs to mutagenic substances of category 2. Pb, Cd, Ni and Hg are also carcinogenic or may be toxic to reproduction. These heavy metals accumulate in human organisms and negatively affect their organs and systems, including the nervous and hematopoietic^31,35,36^. Diisobutyl phthalate (DIBP), dibutyl phthalate (DBP), benzyl butyl phthalate (BBP) and bis(2-ethylhexyl) phthalate (DEHP) are classified as harmful to reproduction category 1B according to the EC Regulation No 1272/2008^34^. Additionally, BBP and DBP phthalates are classified as hazardous to the aquatic environment, according to the EC Regulation No 1272/2008^34^. Dangerous chemicals can get into the human body through the alimentary and inhalation route and through direct contact with the skin^17,25,37,38^. The routes of exposure are related to the nature of the activities performed, i.e. running on the pitch, jumping, slips and falls. These substances can also get into the soil and groundwater^39^. Another problem is that the rubber granules with a particle size of less than 5 mm are classified as a microplastic^40^. This kind of microplastic is removed from sport fields by users or washed away by rainwater. In consequence, it occurs in groundwater and in water flowing out of wastewater treatment plants. After entering the environment, microplastic does not biodegrade but accumulates in the living organisms, including fish and crustaceans, consumed by humans. The release of microplastics contributes to the contamination of ecosystems and food chains. Exposure to microplastics is associated with negative (eco)toxic and physical effects on living organisms. In order to limit the above-mentioned environmental hazards, the work has started on adopting regulations on microplastics, in the European Union since 2016. ECHA’s Risk Assessment Committee (RAC), in June 2020, recommended a ban on microplastics as a filler material in artificial turf pitches after a 6-year transition period^40^. Since 2020 European Commission has been working on developing a proposal to amend the list of substances that are restricted by Annex XVII to the REACH Regulation^41^. In addition, this regulation provides a list of permitted concentrations of PAHs and phthalates. The Supplementary Information includes a detailed description of the European guidelines of the permissible concentrations of chemical substances in materials. Supplementary Tables S1 and S2 present the molecular structures of all of the discussed substances.

The EU requirements for PAHs content in rubber granules used in sport fields will affect the need to increase the quality control of granules in the scope of hazardous substances. If it turns out that the admissible new limits are not complied with, it will be necessary to intensify the search for alternative directions of their application.

The article presents the results of a research work aimed at assessing SBR and EPDM rubber granules from recycling, intended for the construction of sport field surfaces in terms of substances harmful to human health, taking into account their impact on the natural environment. The research was carried out for 17 granules from the surface of sport fields and 67 granules were supplied by recyclers. The scope of the work included the identification of PAH content in rubber granules. From selected specimens leached 18 elements and organotin compounds, as important parameters in assessing the impact on the health of sport fields users, with a broader spectrum than the content of Pb and Cd limited by the requirements of the REACH Regulation.

The PAHs content obtained from the tests was assessed in the light of the requirements of the above-mentioned regulation applicable to rubber granules intended for the construction of sport fields.

The characteristics of the impact of granules on the environment were based on the study of the leachability of PAHs, Pb, Cd, total chromium (Cr) and hexavalent (Cr (VI)), Hg, zinc (Zn) and tin (Sn). In order to achieve the above-mentioned goal, rubber granules used in the construction of sport fields, in which the presence of PAHs was identified through tests, were selected.

## Materials and methods

### Description of test samples and theirs preparation

**Figure 1:**
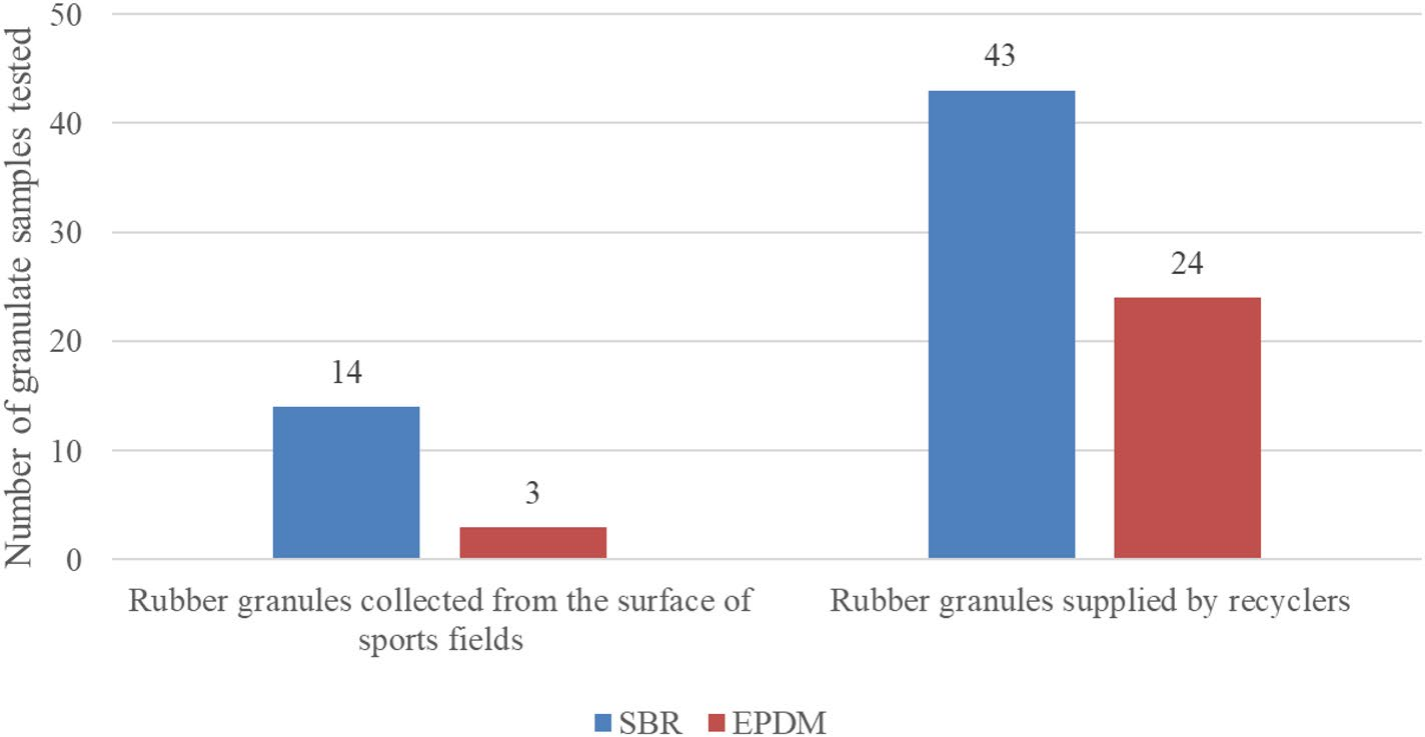
Number of samples of the tested SBR and EPDM granules in relation to their sources of origin.

84 samples of recycled rubber granules with a particle size of 0.5 to 4 mm, produced for the construction of sport field surfaces, were tested. The samples of rubber granules were collected from 17 sport fields and 67 samples rubber granules were supplied by recyclers. Research included 57 samples of SBR granules and 27 samples of EPDM granules. The numbers of samples in relation to their sources of origin are shown in Fig. 1.

**Figure 2:**
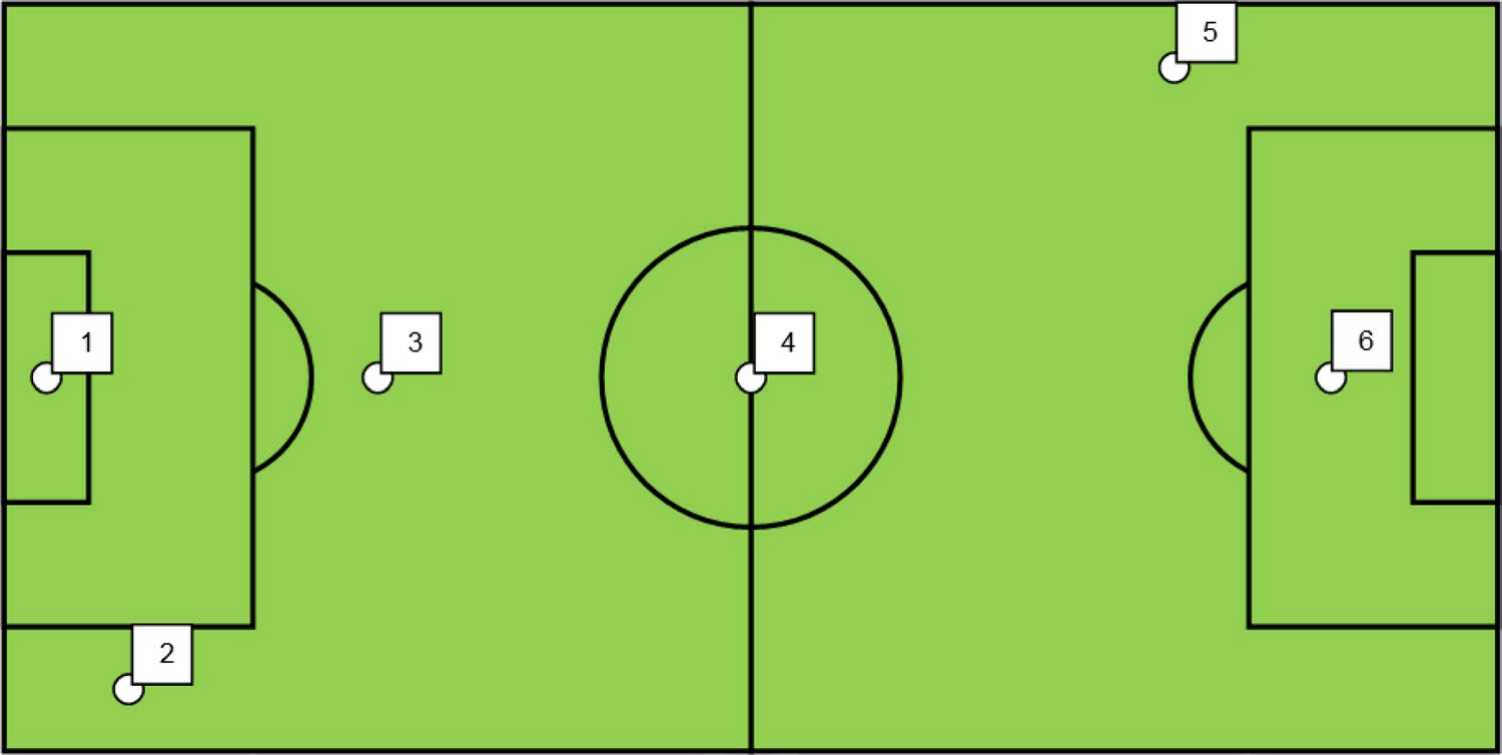
Scheme of distribution of granulate sampling points on a sport field. Designation:  location and numbers of granulate sampling points.

The samples were taken from the surface of sport fields with artificial turf in accordance with the laboratory instructions or delivered to the laboratory by recyclers. The mass of the granulate samples delivered for testing was approx. 0.5 kg. Sampling from sport fields was carried out using a scheme based on 6 sampling points, shown in Fig. 2, in accordance with point 4 of the FIFA guidelines: “Quality Programme for Football Turf. Handbook of Test Methods for Football Turf”. The number and weight of granular samples and the locations of the granular sampling points on the field indicated in the aforementioned guidelines are indicated in order to obtain a representative homogenized granular sample for the tested field^42^.

**Figure 3:**
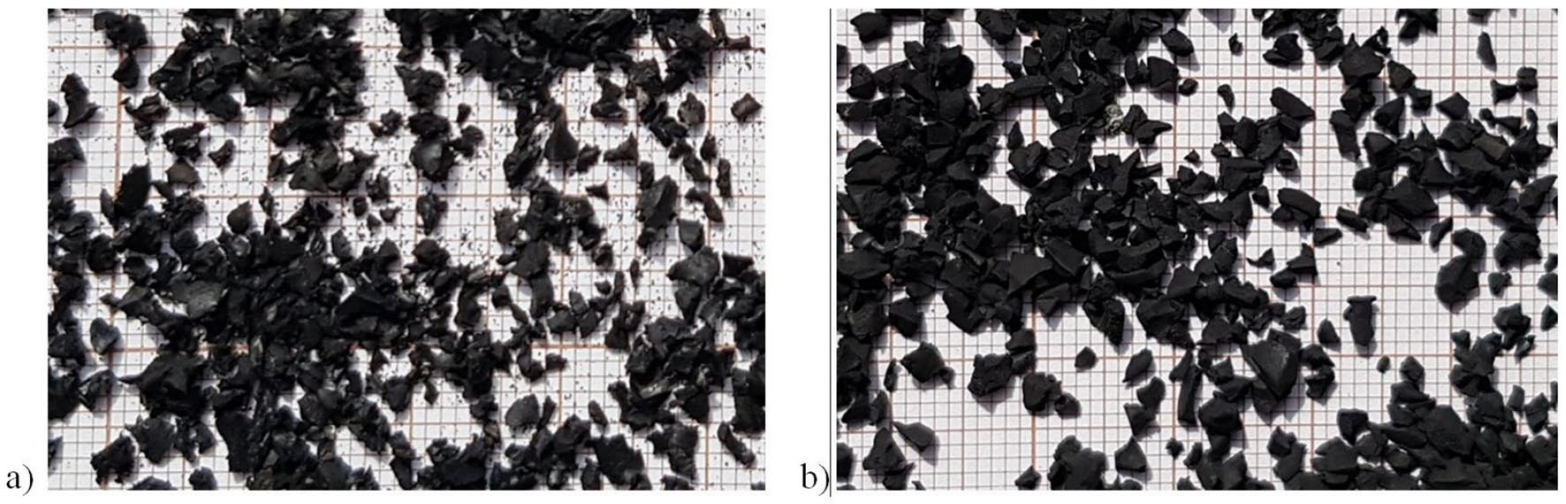
Samples taken from sport fields: (**a**) SBR granules, (**b**) EPDM granules.

At the designated points (1 ÷ 6), 6 samples of granulate were collected. The collected and secured samples were stabilized in the laboratory conditions of natural drying, in which the moisture of the sample was in equilibrium with the ambient moisture. After stabilization, the samples were purified and homogenized to give a pooled sample. Images of exemplary SBR and EPDM granules used in the study are shown in Fig. 3. The average values of the physical parameters of the tested rubber granules are given in Table 1.

**Table 1:** Some physical parameters of the tested SBR and EPDM granules (data from the Technical Data Sheets provided by the recyclers).

Properties	SBR granules	EPDM granules	Test methods
Specific gravity	1.160 kg/m^3^	–	ASTM D1817-05
Bulk density	405 kg/m^3^	500 kg/m^3^	EN 1097-3 or EN ISO 60
Particle size range	0.8–2.0 mm	0.5–2.5 mm	ISO 1322-2 or ISO 2591-1
Particle size < 0.5 mm	0.15%	1.0%	DIN 53477 or ISO 2591-1
Total polymer content (RCH)	45%	–	ISO 9924-3
Ash content	8%	–	ISO 9924-3
Moisture content (Loss: 2 h, 105 °C)	< 1%	–	ASTM D1509
Free metal content	< 0.002%	–	ASTM D5603
Free fibre content	< 0.001%	–	ASTM D5603
Other contamination	< 0.002%	–	ASTM D5603

Samples weighing at least 100 g were taken from the granulate samples using the quartering method. This way allowed to ensure full qualitative and quantitative compliance of the sample composition with the composition of the analyzed material. Samples for testing the content of PAHs were grounded by grinding in a cryogenic mill 6770 Freezer/Mill, by SPEX SamplePrep LLC. Samples for testing other substances were not crushed.

### The scope and methods of testing rubber granules

The scope of the research on rubber granules included: content determination of the PAHs, leached elements, organotin compounds and PAHs. In all samples of rubber granules, the content of 8 polycyclic aromatic hydrocarbons, resulting from the REACH Regulation, was determined: benzo[*a*]pyrene (BaP), dibenz[*a,h*]anthracene (DBAhA), benzo[*e*]pyrene (BeP), benz[*a*]anthracene (BaA), chrysene (CHR), benzo[*b*]fluoranthene (BbFA), benzo[*j*]fluoranthene (BjFA) and benzo[*k*]fluoranthene (BkFA). The content of indeno[1,2,3-*cd*]pyrene (IcdP), benzo[*ghi*]perylene (BghiP), phenanthrene, anthracene, fluoranthene, pyrene and naphthalene was determined for 38 samples from recyclers, additionally, that the number of PAHs covered by the requirements of the document^43^ was increased by 7.

The leaching tests of elements and organotin compounds were carried out for 18 samples and the leachability of PAHs and elements were carried out for 4 samples. The tests were carried out with the methods listed below, using the following apparatus.

The content and leachability of PAHs from rubber granules was determined by gas chromatography with tandem mass spectrometry (GC–MS/MS) using a gas chromatograph coupled with a mass detector GCMS/MS/7890B/7000C. The method was chosen because of the high sensitivity and selectivity obtained for low PAHs levels when used GC–MS/MS, compared to other commonly used analytical techniques such as high-performance liquid chromatography (HPLC) combined with UV, fluorescence or diode array detector (DAD). In studies carried out with the use of the above-mentioned techniques trace amount of PAHs identification is easily interfered by sample matrix and other components if only based on retention^44^.

Determination of leaching of elements: Al, Sb, As, Ba, B, Cd, Co, Cu, Pb, Mn, Hg, Cr, Ni, Se, Sr, Sn, Zn and elution of the Cd, total Cr, Pb, Sn, Zn from rubber granules was carried out by the inductively coupled plasma mass spectrometry (ICP-MS) method with the use of Agilent 7900 ICP-MS (Agilent Technology, Santa Clara, CA, USA). The selected method is characterized by a low limit of quantification, which stands out among other instrumental methods used in elemental analysis, such as ICP-OES or AAS (Inductively coupled plasma–optical emission spectrometry or atomic absorption spectrometry). It is also characterized by high sensitivity and precision, selectivity enabling the simultaneous determination of many elements in complex matrices in a wide range of concentrations.

Leachability of Cr (III) and Cr (VI) and elution of Cr (VI) from rubber granules were determined by high-performance liquid chromatography with inductively coupled plasma mass spectrometry (HPLC-ICP-MS) using Agilent 7700 Series ICP-MS with Agilent 1260 Infinity series HPLC (Agilent Technology, Santa Clara, CA, USA). The decision to use HPLC in conjunction with ICP-MS was dictated by the need to determine chromium in two oxidation states. In the case of the selected method, the speciation separation of Cr (III) and Cr (VI) takes place on the HPLC column, where Cr (III) and Cr (VI) are adsorbed. In the next step it allows for the separation and determination of Cr (III) and Cr (VI) in the ICP-MS spectrometer. The HPLC-ICP-MS method is characterized by a short analysis time and a low detection limit compared to the other spectrophotometric methods used for determination of Cr (VI). The leaching of organotin compounds was assessed on the basis of the results of total Sn leaching.

Cold-vapor atomic absorption spectroscopy (CV-AAS) with the PerkinElmer FIMS 100 mercury analyser was selected for the Hg leaching study due to the use of a unique technique of mercury vapour measurement at room temperature. Among other alternative methods of Hg determination in aqueous solutions (ICP-MS or GF-AAS (graphite furnace atomic absorption spectrometry)), the selected method is distinguished by a low limit of quantification, simple preparation of samples for analysis, easy elimination of interference and short analysis time.

### Tests of the content of PAHs

Shredded samples of rubber granules were subjected to the ultrasonic extraction process for 1 h with the use of toluene as a solvent. Samples were taken from the obtained extract for chromatographic analysis. The analysis was carried out for the following conditions: dispenser operation mode: splitless, carrier gas: Helium: 1.8 ml/min, DB-EUPAH column with dimensions: 20 m × 180 µm × 0.14 µm (the 20 m column is in the form of a coiled wire), injection temperature: 275 °C. The PAHs were identified on the basis of mass spectra and retention times—Table 2.

**Table 2:** Target and identification ions and retention times for the determined PAHs.

Compound	Target ion	Identifying ions
BaP	252	250; 126
DBAhA	278	279; 139
BeP	252	250; 125
CHR	228	226; 113
BbFA	252	250; 126
BaA	228	226; 114
BjFA	252	250; 125
BkFA	252	250; 126
IcdP	276	277; 138
BghiP	276	277; 138
Phenanthrene	178	176; 152
Anthracene	178	176; 152
Fluoranthene	202	200; 101
PYR	202	200; 101
Naphthalene	128	127; 128

### Tests of the leaching of elements and organotin compounds

Samples of rubber granules for the study of the leaching of organotin elements and compounds were extracted in a solution of hydrochloric acid (HCl), with concentration 0.07 ± 0.005 mol/dm^3^ in temperature 37 ± 2 °C. Solutions for the determination the Cr(VI) and Cr(III) prepared by diluting the extraction solution to obtain the pH equal to 7.0 ± 0.5 by adding 1 ml of 0.07 mol/dm^3^ ammonia and 60 µL of 0.1 mol/dm^3^ EDTA solution. In parallel, a reagent blank was prepared, as the test samples were. The obtained extracts were analyzed by ICP-MS and HPLC-ICP-MS. The analyzes were performed for the isotopes of the elements: Al—27, Sb—121, As—75, Ba—137, B—11, Cd—111, 112, Cr—52, 53, Co—59, Cu—63, Pb—206, 207, 208, Mn—55, Hg—201, Ni—60, Se—78, Sr—88, Sn—118, 120, Zn—64, 66.

### Tests of the leachability of polycyclic aromatic hydrocarbons (PAHs) and elements

Determination of the dry mass of the rubber granulate samples for the leachability tests was carried out in accordance with ISO 11465:1999^45^, using a drying oven (Pol-Eco-Apparatus SLW-115 Top, Wodzisław Śląski, Poland) and analytical balances (SARTORIUS, Kostrzyn Wlkp. i Radwag, Radom, Poland).

The rubber granulate samples were dynamically washed with deionized water according to EN 12457-4:2002^46^ providing a ratio of 1 ml of liquid to 1 g of rubber granulate. The pH value of the water used for dynamic leaching did not exceed 6.7. Elution was performed using a bottle/tube roller mixer (Thermo scientific model, Thermo Fisher Scientific (China) Co., Ltd., Shanghai China). After washing, the effluents were left for 15 min and then filtered through 0.45 mm membrane filters using a pressure filtration device.

The leachate obtained from dynamic leaching was subjected to the process of transferring PAHs from the water phase to the organic phase using the algorithm:

- SPE column: C18 bed—6 ml/1000 mg;
- activation: 10 ml of methanol, 10 ml of methanol:water (40:60) (v:v), flow: 1 ml/min;
- sample:eluting solution of methanol (100 ml:10 ml), flow: 0.5 ml/min;
- drying: minimum 15 min, maximum flow;
- elution: 3 × 3 ml of dichloromethane, flow: 0.5 ml/min.

Collected filtrates were evaporated using a vacuum evaporator (IKA RV 05 basic, IKA WERKE GMBH & CO.KG, Staufen) up to 1 ml. Evaporated filtrates were subjected to the chromatographic analysis performed for the conditions as for the determination of PAHs content. The content of eluted PAHs and elements was related to dry mass of the rubber granulate in each sample.

The devices were calibrated and checked on a current basis, including the analysis of control samples, before starting the measurements. Calibrations of the chromatograph, spectrometer and mercury analyzer were performed on solutions of certified reference materials and 2 control samples. The correlation coefficients obtained during the calibration were above 0.995 for all analyzed substances. The analysis of the control samples confirmed the accuracy of the calibration curves, which are the basis for the calculations. Measurements of the content/leachability of the tested substances were carried out for two parallel samples and a reagent blank sample, taking into account the results obtained from it in the analysis of analytical samples. The arithmetic mean of two parallel determinations was assumed as the result of the analytical measurement. Content/leachability conversions of test substances were performed using the GC–MS/MS MassHunter Workstation Software, LCP MHLauncher HPLC-ICP-MS and ACP-MS software and WinLab32 with an AA mercury analyzer FIMS100.

## Results

The conducted tests showed the presence of PAHs in 47 tested rubber granules. Among the granulates with PAHs, 72% were SBR granules and 28% were EPDM granules. The leached elements was found in 44% of the examined granules, including 88% of SBR granules and 12% of EPDM granules. The summarized number of granulate samples, in which the content of PAHs and leached elements were detected in relation to their types and sources of origin, is presented in Table 3.

**Table 3:** Summarized number of granulate samples in which PAHs content and leached elements were detected.

	Number of tested granulate samples		Number of granulate samples where substances were detected		
	SBR	EPDM	SBR	EPDM	
PAHs content	14	3	5	1	Granules from the surface of sport fields
Leached elements	14	3	7	0	
PAHs content	43	24	29	12	Granules supplied by recyclers
Leached elements	0	1	0	1	

### The content of PAHs in rubber granules

Detailed results of the research on PAHs content in granules are presented in Tables 4 and 5. Table 4 shows the results for BaP, BeP, BaA, CHR, BbFA, BjFA, DBAhA and BkFA. Table 5 shows the results for BghiP, naphthalene, phenanthrene, anthracene, IcdP, fluoranthene and PYR. The following abbreviations are used in the tables: SSF—a sample of SBR granules from a sport field, ESF—a sample of EPDM granules from a sport field, SR—SBR granulate sample provided by the recycler, x—average value, U—value of the expanded uncertainty at the confidence level of 95% and the expansion factor k = 2.

**Table 4:** The content of BaP, BeP, BaA, CHR, BbF, BjF, DBahA, BkF in rubber granules.

Sample	Content, mg/kg															
	BaP		BeP		BaA		CHR		BbFA		BjFA		DBAhA		BkFA	
	x	U	x	U	x	U	x	U	x	U	x	U	x	U	x	U
SSF-1	ND	–	5.81	± 2.19	3.94	± 1.09	12.0	± 3.7	ND	–	ND	–	0.96	± 0.30	ND	–
SSF-2	3.15	± 0.89	2.54	± 0.96	4.85	± 1.34	9.96	± 3.15	ND	–	ND	–	0.53	± 0.19	ND	–
SSF-3	2.59	± 0.58	4.90	± 1.83	2.09	± 0.59	8.30	± 2.67	ND	–	ND	–	0.71	± 0.23	ND	–
SSF-4	2.51	± 0.77	4.03	± 1.62	1.56	± 0.56	3.99	± 1.45	2.57	± 1.25	ND	–	ND	–	0.98	± 0.40
SSF-5	4.32	± 0.97	7.67	± 2.85	3.23	± 0.88	8.77	± 3.38	ND	–	ND	–	ND	–	2.36	± 0.89
ESF-1	ND	–	ND	–	ND	–	15.3	± 4.50	ND	–	ND	–	ND	–	ND	–
SR-3	ND	–	ND	–	3.94	± 1.54	10.7	± 3.3	ND	–	ND	–	ND	–	ND	–
SR-4	ND	–	ND	–	ND	–	0.86	± 0.30	ND	–	ND	–	ND	–	ND	–
SR-6	ND	–	12.3	± 4.8	ND	–	ND	-	ND	–	ND	–	21.0	± 6.8	ND	–
SR-12	1.61	± 0.34	2.49	± 0.90	ND	–	5.62	± 1.80	ND	–	ND	–	ND	–	ND	–
SR-13	4.62	± 1.04	6.35	2.37	3.22	± 0.68	9.46	± 3.03	5.20	± 2.18	1.71	± 0.93	ND	–	2.01	± 0.51
SR-14	3.80	± 2.10	6.37	3.56	2.20	± 1.62	8.13	± 7.70	4.16	± 3.93	1.12	± 0.97	ND	–	1.62	± 0.81
SR-15	1.46	± 0.44	2.55	1.04	ND	–	ND	–	ND	–	ND	–	ND	–	ND	–
SR-16	ND	–	ND	–	ND	–	21.1	± 6.8	6.34	± 2.61	0.26	± 0.13	ND	–	ND	–
SR-17	9.24	± 2.02	ND	–	ND	–	ND	–	4.84	± 1.95	1.04	± 0.46	ND	–	ND	–
SR-27	2.88	± 0.81	3.75	1.61	ND	–	8.58	± 2.97	ND	–	ND	–	ND	–	ND	–
Mean	3.62	–	5.34	–	3.13	–	9.44	–	4.62	–	1.03	–	5.81	–	1.74	–
Median	3.02	–	4.90	–	3.23	–	8.77	–	4.84	–	1.08	–	0.84	–	1.82	–
Min	1.46	–	2.49	–	1.56	–	0.86	–	2.57	–	0.26	–	0.53	–	0.98	–
Max	9.24	–	12.29	–	4.85	–	21.12	–	6.34	–	1.71	–	21.03	–	2.36	–

*ND* not detected.

**Table 5:** The content of BghiP, naphthalene, phenanthrene, anthracene, IcdP, fluoranthene and PYR in rubber granules.

Sample	Content, mg/kg													
	BghiP		naphthalene		phenanthrene		anthracene		IcdP		fluoranthene		PYR	
	x	U	x	U	x	U	x	U	x	U	x	U	x	U
SR-3	6.90	± 1.50	1.70	± 070	6.80	± 2.50	7.40	± 1.10	ND	–	11.6	± 1.4	ND	–
SR-13	ND	–	0.22	± 0.11	10.7	± 3.5	0.91	± 0.17	21.8	± 6.5	14.6	± 2.8	29.8	± 7.4
SR-14	ND	–	0.27	± 0.17	10.9	± 3.7	0.72	± 0.15	22.7	± 5.5	11.6	± 5.6	27.4	± 7.3
SR-15	ND	–	ND	–	5.93	± 2.30	0.47	± 0.10	17.8	± 4.4	6.49	± 1.1	22.6	± 4.7
SR-16	21.4	± 6.1	0.77	± 0.34	ND	–	4.06	± 0.73	ND	–	34.3	± 11.6	79.0	± 10.8
SR-17	25.7	± 6.8	1.18	± 0.48	ND	–	ND	–	8.86	± 2.09	ND	–	ND	–
SR-18	9.59	± 2.89	2.71	± 1.05	ND	–	ND	–	ND	–	ND	–	98.3	± 15.9
SR-19	3.85	± 1.17	1.72	± 0.70	ND	–	ND	–	1.36	± 0.46	ND	–	165	± 25
SR-20	1.30	± 0.36	1.78	± 0.85	19.6	± 16.5	20.2	± 17.0	ND	–	ND	–	29.8	± 5.2
SR-21	ND	–	1.62	± 0.66	ND	–	1.63	± 0.27	ND	–	ND	–	ND	–
SR-22	7.32	± 1.79	1.42	± 0.64	ND	–	ND	–	ND	–	8.15	± 1.19	ND	–
SR-23	24.2	± 8.3	1.36	± 0.55	ND	–	ND	–	ND	–	24.4	± 4.9	74.0	± 23.1
SR-24	29.0	± 7.7	1.38	± 0.57	ND	–	ND	–	ND	–	31.8	± 5.3	29.1	± 9.7
SR-25	26.3	± 4.9	1.46	± 0.64	ND	–	5.75	± 1.31	ND	–	41.3	± 14.4	ND	–
SR-26	31.5	± 10.2	1.70	± 0.70	ND	–	9.75	± 2.52	ND	–	53.2	± 21.2	31.5	± 12.2
SR-27	1.93	± 1.20	ND	–	3.24	± 1.32	6.45	± 1.10	1.64	± 1.25	34.7	± 11.3	108	± 34
SR-30	10.6	± 4.0	0.30	± 0.20	20.4	± 3.2	ND	–	ND	–	ND	–	44.6	± 12.8
SR-31	11.7	± 4.9	0.50	± 0.20	19.6	± 5.4	ND	-	ND	–	ND	–	50.8	± 25.3
SR-32	9.60	± 2.2	0.40	± 0.20	ND	–	ND	–	ND	–	16.6	± 3.2	47.2	± 21.0
SR-33	9.60	± 2.2	0.30	± 0.20	ND	–	ND	–	ND	–	19.6	± 2.4	11.7	± 3.9
SR-34	10.3	± 2.3	0.40	± 0.20	8.40	± 2.90	ND	–	ND	–	14.8	± 5.4	44.7	± 14.9
SR-35	10.2	± 2.3	0.40	± 0.20	11.8	± 3.2	ND	–	ND	–	18.6	± 8.3	49.5	± 16.2
SR-40	10.5	± 3.6	0.60	± 0.30	ND	–	ND	–	ND	–	25.7	± 13.8	82.7	± 31.8
SR-41	12.8	± 1.3	ND	–	ND	–	ND	-	ND	–	18.8	± 3.3	65.0	± 23.6
SR-42	ND	–	ND	–	ND	–	ND	–	ND	–	7.61	± 1.22	31.1	± 6.4
SR-43	20.8	± 2.5	ND	–	1.70	± 0.77	3.09	± 0.65	ND	–	24.6	± 5.7	56.1	± 6.2
ER-8	8.30	± 1.8	ND	–	ND	–	ND	–	ND	–	14.7	± 2.8	31.6	± 5.8
ER-10	11.5	± 2.7	ND	–	ND	–	ND	–	ND	–	ND	–	11.9	± 2.8
ER-11	5.58	± 1.31	1.34	± 0.58	ND	–	ND	–	ND	–	6.54	± 1.44	ND	–
ER-12	3.49	± 1.26	1.47	± 0.61	ND	–	ND	–	ND	–	9.40	± 1.04	ND	–
ER-13	ND	–	1.63	± 0.70	1.29	± 0.74	ND	–	ND	–	9.18	± 4.04	ND	–
ER-14	ND	–	1.47	± 0.96	1.57	± 0.61	ND	–	ND	–	9.50	± 1.08	ND	–
ER-15	ND	–	0.99	± 0.49	ND	–	ND	–	ND	–	ND	–	ND	–
ER-16	ND	–	0.90	± 0.46	ND	–	ND	–	ND	–	ND	–	ND	–
ER-17	24.2	± 6.3	0.97	± 0.51	ND	–	5.96	± 1.00	ND	–	53.8	± 14.6	169	± 40
ER-18	21.0	± 7.5	1.31	± 0.41	2.02	± 0.85	2.43	± 0.45	ND	–	ND	–	134	± 22
ER-19	4.00	± 1.28	1.35	± 0.56	ND	–	ND	–	ND	–	6.09	± 1.26	67.3	± 19.7
ER-20	ND	–	1.17	± 0.60	ND	–	ND	–	ND	–	ND	–	ND	–
Mean	13.3	–	1.12	–	8.85	–	5.29	–	12.4	–	20.3	–	61.2	–
Median	10.4	–	1.31	–	7.62	–	4.06	–	13.3	–	15.7	–	48.3	–
Min	1.30	–	0.22	–	1.29	–	0.47	–	1.36	–	6.09	–	11.7	–
Max	31.5	–	2.71	–	20.4	–	20.2	–	22.7	–	53.8	–	169	–

*ND* not detected.

By analysing the results of the tests in Tables 4 and 5, it can be concluded that in 5 SBR granules from sport fields, 4 to 6 types of PAHs were found in the total content ranging from 15.6 to 26.4 mg/kg. The highest levels were recorded for CHR (12.0 mg/kg), BeP (7.70 mg/kg) and BaA (4.30 mg/kg). The BkFA content did not exceed 2.5 mg/kg and the DBAhA did not exceed 1 mg/kg. In the above-mentioned granules no BjFA was found.

In the case of one EPDM sample from sport field, the CHR content was 15.3 mg/kg. No PAH was found in the remaining samples of EPDM granules from sport fields.

In 29 SBR rubber granules, supplied by recyclers, from 1 to 13 types of PAHs were found, in the total content ranging from 0.86 to 172 mg/kg. In the SBR samples were present: PYR (22 samples), BghiP (21 samples) and naphthalene (21 samples), fluoranthene (19 samples). The remaining PAHs types were found in 19 tested granulate samples. PYR (165 mg/kg), fluoranthene (53.2 mg/kg) and BghiP (31.5 mg/kg) were in the highest content in the SBR granules. The lowest content were found for BjFA (less than 1.8 mg/kg). BjFA (less than 1.8 mg/kg) was the lowest content in the SBR granules.

In the case of 12 EPDM rubber granules supplied by recyclers, the presence of 1 to 5 types of PAHs were found in the total content ranging from 0.90 to 254 mg/kg. The most common was naphthalene (10 samples), BghiP (7 samples) and fluoranthene (7 samples). The remaining types of PAHs were found in 7 tested granulate samples. In the highest concentrations was PYR (169 mg/kg) and in the lowest concentrations was naphthalene (less than 2 mg/kg). No presence of BaP, BeP, BaA, CHR, BbFA, BjFA, DBAhA, BkFA, IcdP was found in this kind of granules.

**Figure 4:**
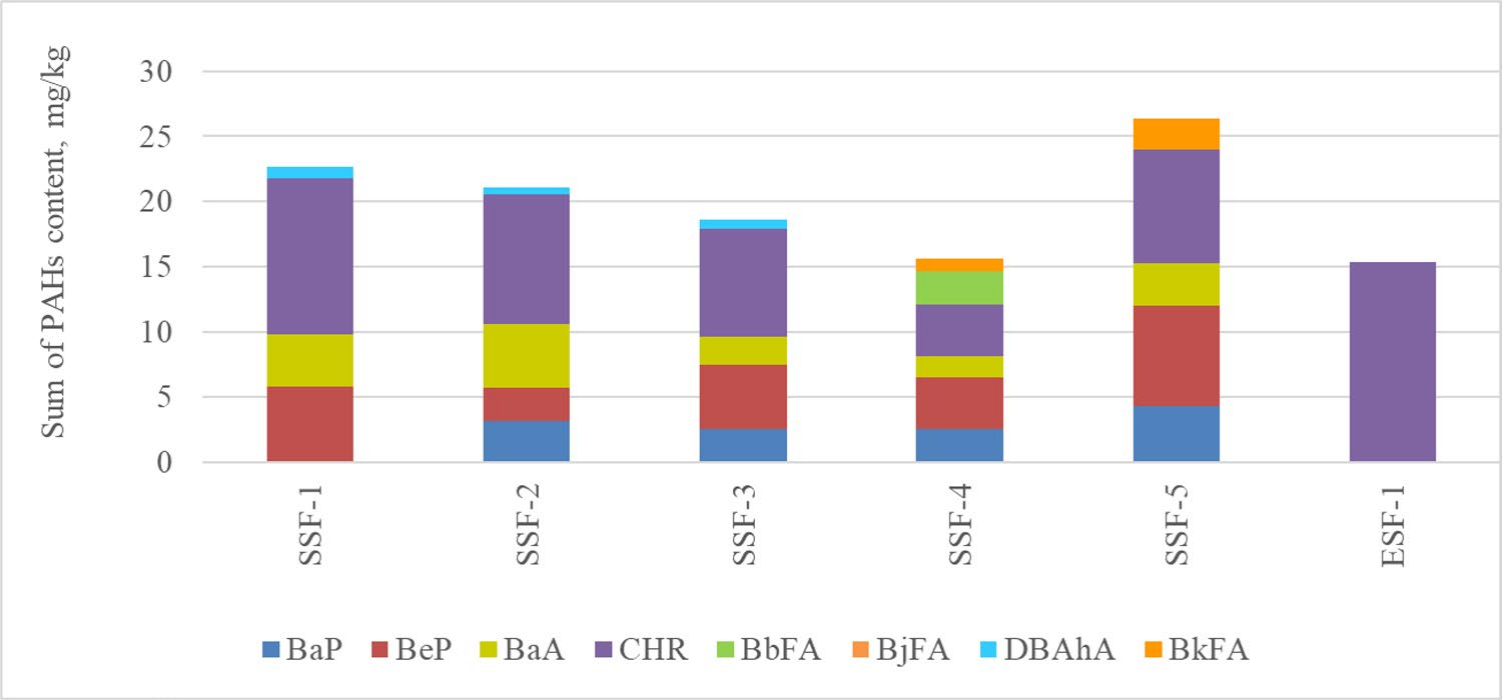
The sum of the PAHs content in the tested rubber granulate samples taken from the sport fields. *SSF* a sample of SBR granules from a sport field, *ESF* a sample of EPDM granules from a sport field.

**Figure 5:**
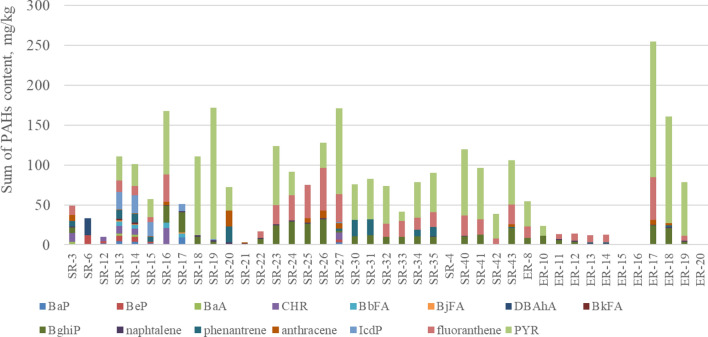
Sum of the PAHs content in the tested samples of rubber granules supplied by recyclers. *SR* SBR granulate sample provided by the recycler, *ER* EPDM granulate sample provided by the recycler.

The total PAHs content in the rubber granulate samples from a sport fields are shown in Fig. 4 and in the rubber granulate supplied by recyclers are shown in Fig. 5.

**Figure 6:**
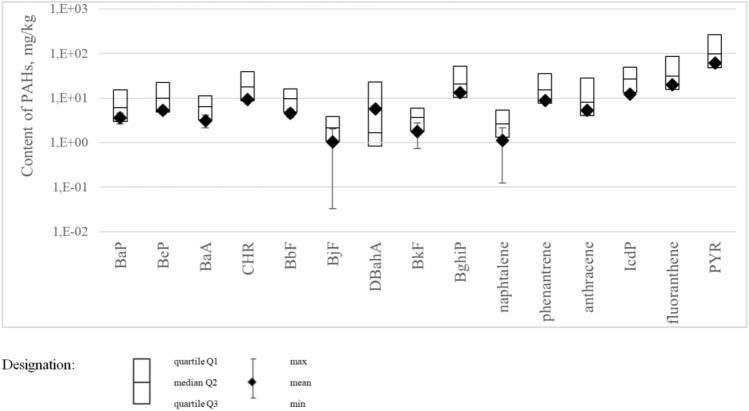
Statistical parameters for the results of tests for PAHs content in rubber granules.

The statistical parameters (median, Q1 and Q3 quartiles, mean, max and min) for the results of testing the PAHs content in rubber granules with a breakdown into particular types of PAHs are presented in Fig. 6. Due to the wide range of obtained results, a logarithmic scale was used.

Comparing the content of PAHs obtained from tests for SBR and EPDM granules from sport fields with the requirements specified in the REACH Regulation, the limit values for plastic and rubber products were exceeded for BaP, BeP, BaA, CHR, BbFA, BjFA, DBAhA and BkFA for 3 samples of SBR granules. When reference was made to the limit values contained in the AfPS GS 2019:01 PAK document, applicable in Germany, exceedances were found for 5 samples of SBR granules and for one sample of EPDM granules.

Comparative analysis of the results of tests for the content of PAHs in samples of SBR granules supplied by recyclers with the requirements specified in the REACH Regulation showed that the sum of BaP, BeP, BaA, CHR, BbFA, BjFA and BkFA was exceeded for 4 samples.

Comparing the PAHs content obtained from the tests for SBR and EPDM granulate samples provided by recyclers with the requirements of the^43^, AfPS GS 2019:01 PAK document showed that the permissible values of the sum of 15 PAHs were exceeded for 28 samples of SBR granules and 10 samples of EPDM granules.

### The elements and organotin compounds

Pb was leached from one sample and Cr (III) was leached from 7 samples out of 14 tested SBR granules from sport fields and one sample of EPDM granules supplied by the recycler. Leached Pb value exceeded 45 mg/kg, and Cr(III) ranged from 0.039 to 0.640 mg/kg. No other elements were washed from the tested granulate samples. The amount of leached organotin compounds did not exceed 6 mg/kg. Detailed results of element leaching tests concerning SBR and EPDM granules are presented in Table 6.

**Table 6:** Results of research on the leaching of elements in SBR and EPDM rubber granules.

Sample	Leaching, mg/kg			
	Pb		Cr(III)	
	x	U	x	U
SSF-1	ND	–	0.10	0.03
SSF-2	45	14	0.11	0.02
SSF-4	ND	–	0.059	0.022
SSF-11	ND	–	0.64	0.08
SSF-12	ND	–	0.17	0.03
SSF-13	ND	–	0.039	0.012
SSF-14	ND	–	0.14	0.09
ER-24	ND	–	0.10	0.05

*SSF* SBR granulate sample from a sport field, *ER* EPDM granulate sample provided by the recycler, *x* average value, *U* expanded uncertainty value with a confidence level of 95% and coverage factor k = 2, *ND* not detected.

Comparing the values of leached elements obtained from the tests for SBR and EPDM granulate samples with the requirements^47^, the permissible value of leached lead was exceeded for one SBR sample from the sport field.

### The PAHs and elements

The study of the leachability of PAHs and elements from rubber granules showed that in the case of 3 samples of SBR granules and one sample of EPDM granules, leached Zn was found. Detailed results of the Zn leachability tests from rubber granules are presented in Table 7.

**Table 7:** Results of leachability tests of Zn from SBR and EPDM rubber granules.

Zn leachability, mg/l	SR40	SR41	SR42	ER17
Mean	0.238	0.266	0.143	0.004
Quantity	3	3	3	3
Median	0.286	0.238	0.107	0.003
Min	0.052	0.234	0.099	0.002
Max	0.375	0.326	0.224	0.006

*SR* SBR granulate sample provided by the recycler, *ER* EPDM granulate sample provided by the recycler.

For the tested three samples of SBR granules and one sample of EPDM granules, no leached remaining 6 elements: Cd, total Cr, Pb, Sn, Cr(VI), Hg was found, and 15 PAHs: BaP, DBAhA, BeP, BaA, CHR, BbFA, BjFA, BkFA, IcdP, BghiP, phenanthrene, anthracene, fluoranthene, PYR and naphthalene was not found, as well as.

Comparing the results of the Zn leaching tests obtained from the tests for SBR and EPDM granulate samples with the requirements^46^, the permissible leaching value was not exceeded.

## Summary and discussion

In order to evaluate the SBR and EPDM recycled rubber granules intended for the construction of sport field surfaces, 84 samples of granules were tested for PAH content, 21% of which were tested for the leaching of organic elements and compounds. Nearly 80% of the samples of granules tested for PAHs content were delivered for testing by recyclers, and the remaining 20% were taken from the surface of sport fields.

### The test results of PAHs content

The tests showed the presence of PAHs in 56% of examined samples of rubber granules, among which the dominant share (34% of the tested samples) were SBR granules supplied by recyclers.

The obtained results of tests for the content of PAHs are consistent with the results of tests carried out by other researchers. The sum of the PAHs content in the tested granules supplied by recyclers reached the highest value of 172 mg/kg, similar to the research conducted by Llompart^24^. Lower values obtained from the tests of rubber granules collected from sport fields correlate with the results obtained by Celeiro^28^ for rubber granules from 15 sport fields, Menichini^23^ for rubber granules from 13 sport fields and Ruffino^48^ for rubber granules from 5 sport fields. In the tested rubber granules used for the surface of sport fields, similarly to the granules tested by Llompart^24^, Menichini^23^ and Ruffino^48^, in the rubber granules from dutch synthetic turf pitches^49^, PYR, naphthalene and fluoranthene occurred most often and in the highest content. Low DBAhA contents were recorded in the granules tested by KOMAG Institute^50^, by Llompart^24^, Menichini^23^ and included in the RIVM report^49^, in contrast to the granules tested by Ruffino^48^, where the DBAhA content was up to 8.13 mg/kg. The research of KOMAG Institute, as well as the research carried out by Menichini^23^ showed a wide range of PAHs content, regardless the type and source of granules. The PAHs content in rubber granules depends on many factors, i.e. the quality of recycled waste, the duration of use of the granulate on the pitch, the frequency of its replacement and the type of material used for this purpose, according to Ref.^23^.

The high PAHs content in granules from sport fields is particularly important due to the fact that they are used by children who are directly exposed to the granules while running on the pitch, jumping, slipping and falling. It should be noted that the physical activity of children on sport fields increases the rate of their metabolism, which translates into the intensity of absorption of toxic chemicals from the environment and may, consequently, lead to threats to their health. They are also shorter than adults, so they have easier access to these compounds.

### The results of elements and organotin compounds leachability

The study on the elements and organotin compounds leaching from the granules used for sport fields showed a high amount of Pb leached from the granules in one case. The remaining examined elements were not extracted from the granules. These results differ from the results obtained by FA Group for SBR granules from artificial turf, which showed leached Zn at the level of 365 mg/kg, Cu at the level of 41 mg/kg and Al at the level of 6.15 mg/kg. The cause may be the lack of information about the source of the granules tested by FA Group^17^.

Taking into account that the permissible value of the leached Pb was exceeded for statistically 5% of the tested granules, it can be concluded that the study on the elements leaching should be an important feature of the safety assessment of rubber granules used for the surface of sport fields.

### The results of PAHs and elements leaching tests

The leaching tests of rubber granules used for sport fields showed small amounts of leached Zn for all of 3 SBR granules and 1 EPDM granulate specimen. The remaining 6 elements and 15 PAHs were not washed out from the tested rubber. The above results of the PAHs and elements leachability consistent with the results obtained by Gomes^22^ and Bocca^20^ for car tire granulates. The elution of Zn and the lack of elution of other elements from rubber granules from sport fields were also found by Kleps^33^, Niesłochowski and Deptuła^26^ and Nilsson^18^. In the case of tests carried out by Bocca^20^ and by Kleps^33^, the leaching of Zn was determined for two types of environmental conditions, with the use of deionized water and under acidic conditions (pH: 5). The leaching liquid with the reduced pH increased the leachability of zinc. It increased also leachability of Cd, Cr and Pb^20^.

On the basis of the obtained test results, it was found that the rubber granules do not pose a threat to the natural environment due to the leaching of PAHs and elements. However, taking into account the size of rubber granules, which determines their classification as microplastics, polluting ecosystems and food chains. The content of PAHs is important due to the threats to living organisms.

## Conclusions

Rubber waste, including waste from used car tires, is a significant and problematic stream of polymer waste in terms of quantity. They are used for the production of rubber granules for the surface of sport fields, among others. The assessment of recycled SBR and EPDM rubber granules, carried out as part of the study, showed that they contain harmful to human health PAHs in amounts exceeding the permissible limits. Extender oils and black carbon used in the manufacture of tires can be the source of PAHs in SBR granules^51–54^. This state of affairs is confirmed by the results of research conducted by other global research units and the new limitation in this regard introduced by the European Commission in July 2021, in the REACH Regulation. On the other hand, the preliminary studies on leaching, using methods used to assess the impact of waste on the environment carried out by the authors of this publication, showed that no harmful elements and PAHs were washed out from the tested rubber granules. In the light of literature reports on the increase of leached metals after using a liquid with lowered pH, equal to 5, it can be concluded that the standardized method of leaching the above-mentioned with the deionized water does not reflect the actual leaching conditions in the environment in which the rubber granules are embedded. This method requires further development in terms of adjusting the parameters of the leaching liquid to them.

The results of the presented research may constitute the basis for undertaking work on the development of the application of rubber granules alternative to the surface of sport fields. In further works, the authors intend to analyze the possibility of reducing the risk associated with contamination of ecosystems and food chains with rubber granulate particles with an increased content of PAHs. There will be attempts to exemplify their application in construction or hydro construction. They are known to be used in bridge abutments, road embankments, reinforcing soil and roadsides, asphalt pavements, making retaining walls, vibro-insulation boards, soundproofing insulations, damping screens and paving stones^11,55–61^.

## Supplementary Information

Supplementary Information.
