# Supplementary material for: Tests of rubber granules used as artificial turf for football fields in terms of toxicity to human health and the environment
Source: Sci Rep. 2022 Apr 23;12:6683. doi: 10.1038/s41598-022-10691-1 (PMC9035180; doi:10.1038/s41598-022-10691-1)
Supplement: Supplementary file 1 — Supplementary Information. [file 41598_2022_10691_MOESM1_ESM.docx]

**Supplementary Information**

**Tests of rubber granules used as artificial turf for football fields
in terms of toxicity to human health and the environment**

Beata Grynkiewicz-Bylina^1^, Bożena Rakwic^1^, Barbara Słomka-Słupik^2,*^

^1^ KOMAG Institute of Mining Technology, Laboratory of Material Engineering and Environment, 44-101 Gliwice, Poland; bbylina@komag.eu; brakwic@komag,eu

^2^ Silesian University of Technology, Faculty of Civil Engineering, 44-100 Gliwice, Poland

^*^ Correspondence: [barbara.slomka-slupik@polsl.pl](mailto:barbara.slomka-slupik@polsl.pl)

**The guidelines of the permissible concentrations of chemical substances in materials.**

According to the guidelines of the International Football Federation (FIFA) entitled "Handbook of Requirements. FIFA Quality Program for Football Turf"^1^, sport field surfaces must not contain chemicals, which may show toxic, carcinogenic, mutagenic or toxic effects on reproduction, in contact with human skin.

The requirements specifying the permissible concentrations of chemical substances in rubber granules are included in the REACH^2^. In the case of heavy metals, their permissible content in granules is 0.01% by weight for cadmium (Cd) and 0.05% by weight for lead (Pb). In the case of phthalates permissible content in granules is 0.1% by weight for bis(2-ethylhexyl) (DEHP), dibutyl (DBP), benzyl butyl (BBP), diisobutyl (DIBP), in relation to the weight of the material with plasticizers.

A more complex situation concerns the limit values for PAHs content. The current limits apply to the content of these compounds in plastic and rubber products that may come into direct contact with the human skin or oral cavity. Theirs amount should be less than 1 mg/kg for benzo[*a*]pyrene (BaP), dibenz[*a,h*]anthracene (DBAhA), benzo[*e*]pyrene (BeP), benz[*a*]anthracene (BaA), chrysene (CHR), benzo[*b*]fluoranthene (BbFA), benzo[*j*]fluoranthene (BjFA) and benzo[*k*]fluoranthene (BkFA).

In the case of mixtures of chemical substances, the limits of the above-mentioned chemical compounds are determined to the level of 100 mg/kg for BaP and DBAhA, to 1000 mg/kg for BeP, BaA, CHR, BbFA, BjFA and BkFA. According to the interpretation of the European Commission, presented in Item 2.1, in Annex 1 of the European Chemical Agency "*Guideline on the scope of restriction entry 50 of Annex XVII to REACH: Polycyclic aromatic hydrocarbons in articles supplied to the general public*"^3^, synthetic surfaces on sport fields, available to the public, regardless of the type of their ownership (public or private), in the light of the applicable regulations, should be treated as the final products^3^. On July 21, 2021, the European Commission published a Regulation 2021/1199^4^ in which, from August 10, 2022, the requirements for PAHs content are tightened. A limit of the content of the sum of eight PAHs at the level of 20 mg/kg in rubber granules used as filling material on artificial turf fields or poured loose on playgrounds was introduced.

Additional restrictions regarding PAHs applied in Germany by Product Safety Commission is located in the document AfPS GS 2019:01 PAK^5^. In this document, the number of restricted PAHs was increased by the following 7 hydrocarbons: indeno[1,2,3-*cd*]pyrene (IcdP), benzo[*ghi*]perylene (BghiP), phenanthrene, anthracene, fluoranthene, pyrene (PYR), naphthalene. The limit values were also tightened in the content of chrysene, BaA, BbFA, BkFA, BjFA, BaP, BeP, IcdP, DBAhA, BghiP in plastic products, with which children have contact with up to the level of 0.2 mg/kg in relation to the weight of the material with the addition of PAHs. The limit values were also tightened in the content of the sum of phenanthrene, anthracene, fluoranthene, and PYR up to the level of 1 mg/kg, the content of naphthalene – up to the level of 1 mg/kg and the sum of the content of the aforementioned 15 PAHs – up to the level of 1 mg/kg.

In the case of surfaces of sports fields and playgrounds, of which children are the users, the toxicity assessment is extended to the requirements for category III of materials, what is included in standard^6^ for the leaching of 18 elements: aluminum (Al), antimony (Sb), arsenic (As), barium (Ba), boron (B), cadmium (Cd), trivalent chromium (Cr III), hexavalent chromium (Cr VI), cobalt (Co), copper (Cu), lead (Pb), manganese (Mn), mercury (Hg), nickel (Ni), selenium (Se), strontium (Sr), tin (Sn), zinc (Zn) and 10 organotin compounds: dibutyl tin (DBT), tributyl tin (TBT), tetrabutyl tin (TeBT), monoctyl tin (MOT), dioctyl tin (DOT), dipropyl tin (DProT), diphenyl tin (DPhT), triphenyl tin (TPhT), methyl tin (MeT), butyl tin (BuT)^6,7^. The permissible values of leaching of elements and organotin compounds for category III materials (in accordance with^6^) are: 28,130 mg/kg for Al, 560 mg/kg for Sb, 47 mg/kg for As, 18,750 mg/kg for Ba, 15,000 mg/kg for B, 17 mg/kg for Cd, 460 mg/kg for Cr (III), 0.053 mg/kg for Cr (VI), 130 mg/kg for Co, 7,700 mg/kg for Cu, 23 mg/kg for Pb, 15,000 mg/kg for Mn, 94 mg/kg for Hg, 930 mg/kg for Ni, 460 mg/kg for Se, 56,000 mg/kg for Sr, 180,000 mg/kg for Sn, 46,000 mg/kg for Zn and 12 mg/kg for the sum of organotin compounds. The mentioned standard**^6^** is dedicated to the testing of toys and is also recommended for use in the assessment of chemical hazards related to the use of children's articles, in accordance with the Guide CEN/TR 13387-2:2018 "Child care articles. General safety guidelines. Part 2. Chemical hazards"^8^.

The impact of rubber granules from the surface of sport fields and playgrounds on the natural environment is determined in accordance with the DIN 18035-6 and DIN 18035-7 standards^9,10^, and assessed in the light of their limits for content in the water extract: dissolved organically bound carbon (DOC), extractible organic halides (EOX), Pb, Cd, total Cr and Cr (VI), Hg, Zn, Sn, and in the case of recycled granulates other than waste tires, also chlorinated paraffins and phthalates. The permissible values of the leached elements are: 0.025 mg/dm^3^ for Pb, 0.005 mg/dm^3^ for Cd, 0.05 mg/dm^3^ for total Cr, 0.008 mg/dm^3^ for Cr (VI), 0.001 mg/dm^3^ for Hg, 0.5 mg/dm^3^ for Pb and 0.04 mg/dm^3^ for Sn.

**Table S1.** Structure of restricted phthalates.

| **Name** | **Abbreviation** | **CAS no.** | **Molecular structure** |
| --- | --- | --- | --- |
| bis(2-ethylhexyl) phthalate | DEHP | 117-81-7 | 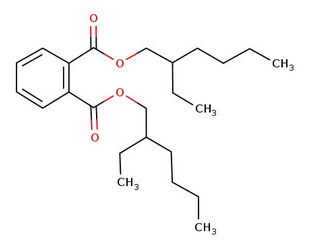 |
| dibutyl phthalate | DBP | 84-74-2 | 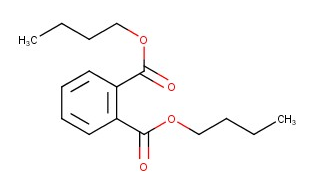 |
| benzyl butyl phthalate | BBP | 85-68-7 | 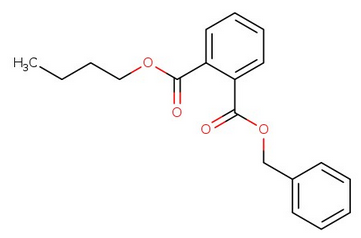 |
| diisobutyl phthalate | DIBP | 84-69-5 | 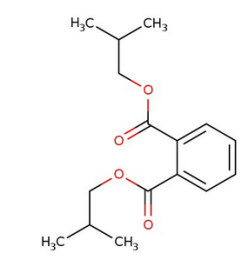 |

**Table S2.** Structure of restricted PAHs.

| **Name** | **Abbreviation** | **CAS no.** | **Molecular structure** |
| --- | --- | --- | --- |
| benzo[*a*]pyrene (benzo[def]chrysene) | BaP | 50-32-8 | 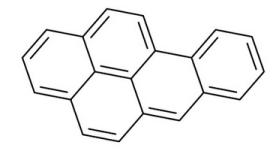 |
| dibenz[*a,h*]anthracene | DBAhA | 53-70-3 | 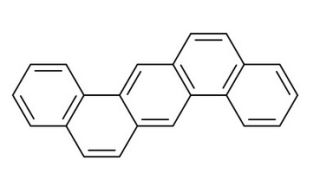 |
| benzo[*e*]pyrene | BeP | 192-97-2 | 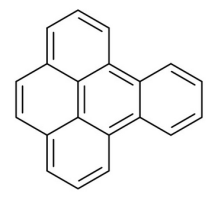 |
| benz[a]anthracene | BaA | 56-55-3 | 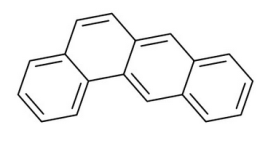 |
| chrysen | CHR | 218-01-9 | 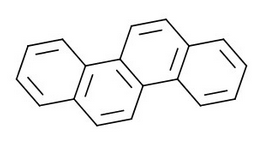 |
| benzo[*b*]fluoranthene (benzo[e]acephenanthrylene) | BbFA | 205-99-2 | 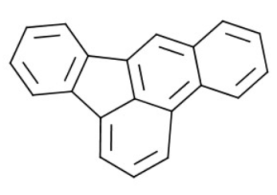 |
| benzo[*j*]fluoranthene | BjFA | 205-82-3 | 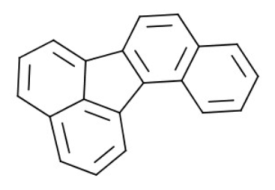 |
| benzo[*k*]fluoranthene | BkFA | 207-08-9 | 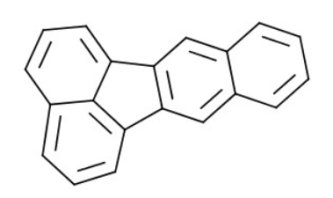 |
| indeno[1,2,3-*cd*]pyrene | IcdP | 193-39-5 | 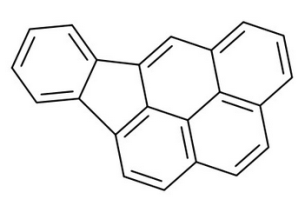 |
| benzo[*ghi*]perylene | BghiP | 191-24-2 | 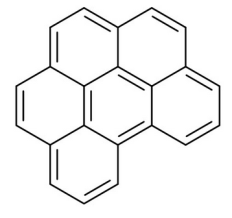 |
| phenanthrene | - | 85-01-8 | 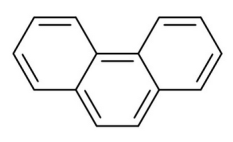 |
| anthracene | - | 120-12-7 | 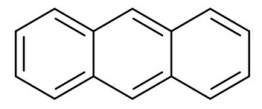 |
| fluoranthene | - | 206-44-0 | 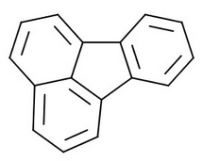 |
| pyrene | PYR | 129-00-0 | 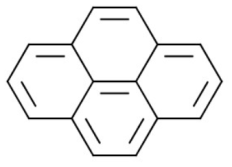 |
| naphthalene | - | 91-20-3 | 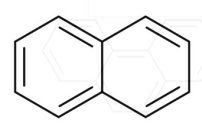 |

**References**

1. FIFA, Quality Programme for Football Turf. Handbook of Requirements. October 2015 Edition, https://football-technology.fifa.com/media/1239/fqp-handbook-of-requirements-2015-v31-w-cover.pdf (2015).
2. European Parliament, Regulation (EC) No 1907/2006 of the European Parliament and of the Council of 18 December 2006 concerning the Registration, Evaluation, Authorisation and Restriction of Chemicals (REACH), establishing a European Chemicals Agency, amending Directive 1999/45/EC and repealing Council Regulation (EEC) No 793/93 and Commission Regulation (EC) No 1488/94 as well as Council Directive 76/769/EEC and Commission Directives 91/155/EEC, 93/67/EEC, 93/105/EC and 2000/21/EC. OJ L 396, 30.12.2006, p. 1-849 with further amendments, https://eur-lex.europa.eu/legal-content/EN/TXT/HTML/?uri=CELEX:02006R1907-20210215&qid=1624208846161&from=EN (2021).
3. ECHA, Guideline on the scope of restriction entry 50 of Annex XVII to REACH: Polycyclic aromatic hydrocarbons in articles supplied to the general public. European Chemicals Agency, https://echa.europa.eu/documents/10162/15792/guideline_entry_50_pahs_en.pdf/f12ac8e7-51b3-5cd3-b3a4-57bfc2405d04 (2018).
4. European Parliament, Commission Regulation (EU) 2021/1199 of 20 July 2021 amending Annex XVII to Regulation (EC) No 1907/2006 of the European Parliament and of the Council as regards polycyclic-aromatic hydrocarbons (PAHs) in granules or mulches used as infill material in synthetic turf pitches or in loose form on playgrounds or in sport applications. OJ L 259, 21.7.2021, p. 1-5, https://eur-lex.europa.eu/legal-content/EN/TXT/HTML/?uri=CELEX:32021R1199&from=EN (2021).
5. Product Safety Commission (AfPS) GS Specification, Testing and assessment of polycyclic aromatic hydrocarbons (PAHs) in the course of awarding the GS mark - Specification pursuant to article 21(1) no. 3 of the Product Safety Act (ProdSG). AfPS GS 2019:01 PAK. https://www.baua.de/DE/Aufgaben/Geschaeftsfuehrung-von-Ausschuessen/AfPS/pdf/AfPS-GS-2019-01-PAK-EN.pdf?__blob=publicationFile&v=4 (2020).
6. European Committee for Standardization, EN 71-3:2019+A1:2021 Safety of toys - Part 3: Migration of certain elements (2021).
7. ECHA, Opinion on an Annex XV dossier proposing restrictions on intentionally-added microplastics. Committee for Risk Assessment (RAC), Committee for Socio-economic Analysis (SEAC). ECHA/RAC/RES-O-0000006790-71-01/F, ECHA/SEAC/RES-O-0000006901-74-01/F. European Chemicals Agency, 20 December 2020, https://echa.europa.eu/documents/10162/a513b793-dd84-d83a-9c06-e7a11580f366 (2020**)**.
8. European Committee for Standardization, CEN/TR 13387-2:2018 Child care articles. General safety guidelines. Part 2. Chemical hazard (2018).
9. German Institute for Standardization, DIN 18035-6:2021-08 Sports grounds - Part 6. Synthetic surface (2021).
10. German Institute for Standardization, DIN 18035-7:2019-12 Sports grounds - Part 7. Synthetic turf areas (2019).
